# Shape matters: morphological metrics of glioblastoma imaging abnormalities as biomarkers of prognosis

**DOI:** 10.1038/s41598-021-02495-6

**Published:** 2021-12-01

**Authors:** Lee Curtin, Paula Whitmire, Haylye White, Kamila M. Bond, Maciej M. Mrugala, Leland S. Hu, Kristin R. Swanson

**Affiliations:** 1 Mathematical Neuro-Oncology Lab, Precision Neurotherapeutics Innovation Program, Department of Neurological Surgery, Mayo Clinic, 5777 E Mayo Blvd, Phoenix, AZ 85054 USA; 2 Mayo Clinic School of Medicine, Rochester, MN USA; 3 Department of Neurology, Mayo Clinic, 5777 E Mayo Blvd, Phoenix, AZ 85054 USA; 4 Department of Radiology, Mayo Clinic, 5777 E Mayo Blvd, Phoenix, AZ 85054 USA

**Keywords:** Cancer imaging, CNS cancer, Applied mathematics

## Abstract

Lacunarity, a quantitative morphological measure of how shapes fill space, and fractal dimension, a morphological measure of the complexity of pixel arrangement, have shown relationships with outcome across a variety of cancers. However, the application of these metrics to glioblastoma (GBM), a very aggressive primary brain tumor, has not been fully explored. In this project, we computed lacunarity and fractal dimension values for GBM-induced abnormalities on clinically standard magnetic resonance imaging (MRI). In our patient cohort (n = 402), we connect these morphological metrics calculated on pretreatment MRI with the survival of patients with GBM. We calculated lacunarity and fractal dimension on necrotic regions (n = 390), all abnormalities present on T1Gd MRI (n = 402), and abnormalities present on T2/FLAIR MRI (n = 257). We also explored the relationship between these metrics and age at diagnosis, as well as abnormality volume. We found statistically significant relationships to outcome for all three imaging regions that we tested, with the shape of T2/FLAIR abnormalities that are typically associated with edema showing the strongest relationship with overall survival. This link between morphological and survival metrics could be driven by underlying biological phenomena, tumor location or microenvironmental factors that should be further explored.

## Introduction

Glioblastoma (GBM) is an aggressive and highly infiltrative primary brain tumor with a median survival of only 15–16 months with standard-of-care treatment^1–3^. Due to the sensitive location of the tumor, opportunities for biopsies are limited and there is a heavy reliance on imaging, typically magnetic resonance imaging (MRI), to assess the severity and progression of the disease. Morphology has been connected to tumor diagnosis, aggressiveness and prognosis in a variety of cancers^4–12^. There remains a relative lack of studies on the prognostic implications of these shape metrics of GBM-induced abnormalities on MRI. There are three key GBM-associated regions detectable on standard-of-care MRI. The first is the enhancing region present on T1-weighted MRI with gadolinium contrast (T1Gd MRI), caused through leakage of gadolinium across disrupted vasculature^13^. This enhancement typically spatially correlates with the bulk of the tumor, particularly in a pretreatment setting^14^. The second region is necrosis, caused by a lack of sufficient nutrients and necrosis-inducing factors, typically present as a central hypointense region on T1Gd MRI surrounded by enhancement. The third is the abnormal region present on both T2-weighted and fluid attenuated inversion recovery (FLAIR) MRIs that spatially correlates to edema and infiltrative tumor cells^15^.

In this work, we focus on the prognostic impact of two morphological metrics that quantify these GBM regions. The first is fractal dimension, a measure of the consistency of a shape with itself at varying spatial scales. If a shape is extremely self-consistent, it will have a high fractal dimension. Lacunarity is a quantifiable measure of how shapes fill space, and more generally considers heterogeneity. Higher lacunarity values occur in shapes that are disconnected and more heterogeneous. There are many examples of fractal dimension and lacunarity providing clinical insight in oncology. For example, fractal dimension and lacunarity have been shown as prognostic markers for melanoma and laryngeal carcinoma^16,17^. Differences in fractal dimension between healthy and pathological tissue have been found in renal chromophobe carcinoma^18^. Fractal dimension has been shown to distinguish benign and malignant breast tumors in both digitized histology^5^ and ultrasound images^19^. There is also a wealth of morphological studies on lung cancers^20^. Within glioma, the lacunarity of T2-weighted MRI abnormalities has been shown to distinguish glioma grade^21^ and the fractal dimension of brain vasculature in susceptibility-weighted imaging has been shown to distinguish brain tumor grade^22,23^. A separate study of 95 patients has found fractal dimension and lacunarity, applied to pretreatment necrotic regions present on T1Gd MRIs can distinguish overall survival (OS) and progression free survival (PFS) in GBM^7^. We seek to build on the results of this previous study on a larger cohort through the inclusion of analyses on other MRI abnormalities. We will also look for correlations between these morphological metrics with patient age, patient sex and imaging abnormality volumes.

In a retrospective cohort of 402 patients with primary GBM, we have calculated lacunarity and fractal dimension values of imaging abnormalities using T1Gd MRI and T2/FLAIR images. We find statistically significant relationships between these morphological metrics applied to imaging abnormalities and survival metrics in patients with GBM, both for OS and PFS.

## Methodology

### Patient cohort

We queried our multi-institutional database of retrospective patient data for patients with first-diagnosed GBM. We required these patients to have available segmented pretreatment T1-weighted MRI with gadolinium contrast in our database, as well as age at diagnosis, sex and overall survival (confirmed death, alive, or lost to follow-up). We also required that these patients’ tumors did not contain a significant cystic component, which typically presents as hypointense with surrounding enhancement and smooth on T2-weighted MRI. Cystic components are typically round and may provide a survival benefit to patients^24^, which may have interfered with relationships between morphological metrics and survival. Hypointense cystic fluid would also interfere with our ability to consistently capture necrotic regions, which are also hypointense. This resulted in a cohort of 402 patients. Where available, we also noted progression-free survival (n = 125), extent of resection (n = 274) and stored T2/FLAIR segmentations (n = 257). As many of our patients were diagnosed before the current standard of care (SOC) protocol was established, our cohort consists of a variety of treatment protocols. We have established a subcohort of 142 patients known to have received the current SOC, and refer to these as “current SOC patients”. See Table 1 for further breakdowns of cohort sizes across different imaging regions, and Supplement 1 for a sex-specific breakdown of these groups.

**Table 1:** Cohort of patients with known overall survival (OS) and progression free survival (PFS).

	T1Gd MRI		T2/FLAIR MRI
	Necrosis	Enhancement with necrosis	Edema
**All patients**			
OS	390	402	257
PFS	125	130	78
**Current SOC**			
OS	135	142	92
PFS	86	89	56

This table shows the number of patients with available imaging ROIs and known OS and PFS, including the subsets known to have received the current standard of care (SOC). The discrepancy between patients with available necrosis ROIs and T1Gd enhancing ROIs is due to 12 patients with negligible necrosis that precluded addition of those tumors to the necrosis-specific analysis.

### Biomedical imaging and ROI segmentation

Using pretreatment T1Gd MRI, enhancing abnormalities were segmented by trained individuals and necrotic regions were segmented using the segmentations of T1Gd enhancement within an automated dilation erosion algorithm. T2/FLAIR abnormalities were also segmented by trained individuals. We use an in-house thresholding-based segmentation software to create segmentations. Segmentation regions were used alongside image dimensions to calculate imaging abnormality volumes (cm^3^). We also use segmented volumes to compute radii of equivalent spherical volumes (cm). The resolution of each image was stored. There are three different segmentations used in this analysis, the first is necrotic regions, the second is T1Gd enhancing regions with necrotic regions, and the third is abnormal regions on T2/FLAIR MRI associated with edema. We chose to include the necrotic regions with the enhancement on T1Gd MRI to avoid the conflation of necrosis outlines that would otherwise be present in T1Gd-enhancing regions alone.

### Lacunarity and fractal dimension

**Figure 1:**
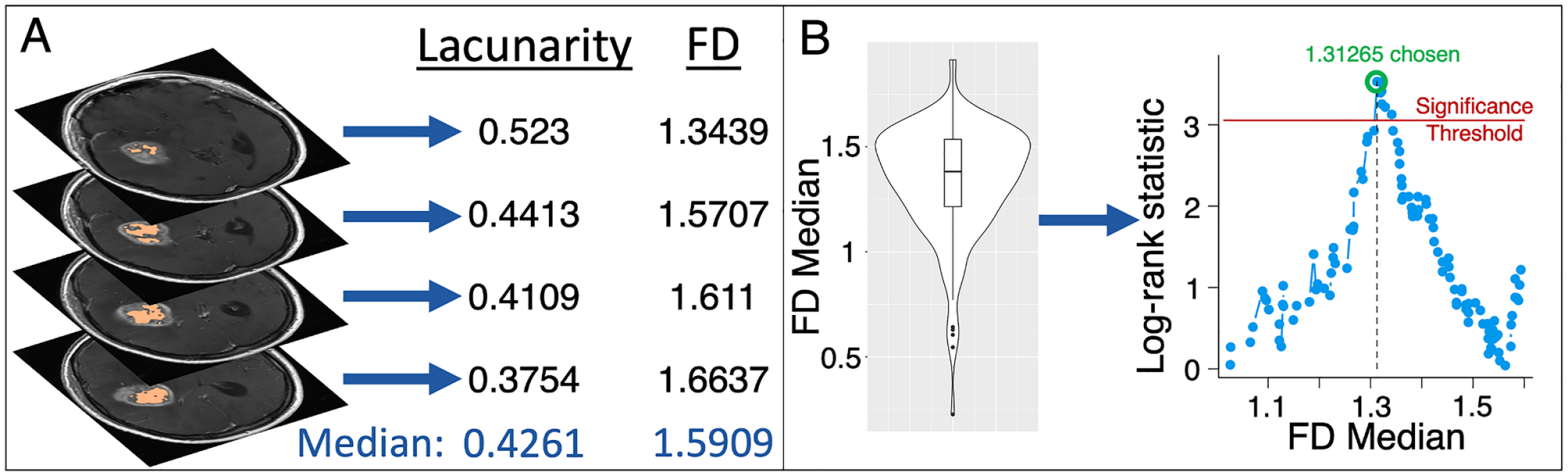
Computing lacunarity and fractal dimension (FD) and testing the statistical significance of these against patient survival data. (**A**) As an example, lacunarity and FD are computed on each slice of T1Gd necrosis segmentations. The median of these values is stored, giving one value of lacunarity and FD for each patient. We do the same for T1Gd enhancement with necrosis, and T2/FLAIR edematous regions. (**B**) Individual median values are collated into a cohort analysis. Each median value that does not split the cohort into groups of less than 10% of the cohort size is then tested as a cutoff to distinguish either overall survival or progression free survival. A log-rank test provides a significance value for each potential cutoff and a log-rank statistic is calculated alongside a separate significance threshold that accounts for multiple comparisons. The maximal log-rank statistic is chosen as this maximally distinguishes the two groups. This is carried out for lacunarity and FD, across necrosis, enhancement with necrosis, and edema regions.

We used the FracLac plugin for ImageJ to calculate lacunarity and fractal dimension values for each 2D segmentation^25,26^. Image slices with segmentations totaling 5 pixels or less were excluded. The FracLac software uses a box-counting algorithm to compute lacunarity and fractal dimension; we used a minimum of 2 pixels and a maximum of 45% of each MRI slice for these box sizes. To compute fractal dimension, grids with varying box sizes are placed over a region, and the number of boxes needed to cover the region in question is recorded for each grid. The log of this number is then plotted against the log reciprocal of the length of each box and the gradient of the regression line for this plot is the fractal dimension. For lacunarity, a similar process is undertaken with the numbers of pixels in each box recorded as a distribution for each box length, with standard deviation σ and mean μ. Lacunarity is then the mean over all box sizes of (σ/μ)^2^. Due to the dependency of fractal dimension and lacunarity on grid placement, we used a mean fractal dimension and a mean lacunarity calculated over 12 grid placements for each 2D segmentation. We then stored the following metrics for both fractal dimension and lacunarity across MRI slices, to give one value of each per pretreatment MRI: the median value, the mean value, the range of values and the variance of values. Although previous work by others has shown survival differences with mean values^7^, we saw clearer signals with median values, as these were less impacted by outliers. We present results of mean values in Supplement 2 to compare more closely to previously published work^7^. In Fig. 1A, we present a schematic of how we compute lacunarity and fractal dimension values. For a subset of images with multiple segmentations available (necrosis = 201, enhancement with necrosis = 211, edema = 136), we tested the intraclass coefficients (ICCs) of lacunarity and fractal dimension and found very good agreement between segmentations (lowest ICC 0.898). We present details of this analysis in Supplement 3.

### Statistical analyses

We used log-rank tests to ascertain the significance in overall survival and progression-free survival differences in our cohort; we use Kaplan–Meier curves to visualize these differences. All the analysis presented here was carried out in R^27–31^. Throughout this work, we set a p-value threshold of 0.05, below which we consider our results to be statistically significant. As this work uses multiple comparisons, we have used the maxstat package in R to adjust our p-values appropriately^32^. Namely, we have implemented the adjustment method first presented by Lausen and Schumacher^33^. We use the chosen threshold to divide our cohorts into two groups: the first consists of patients with values lower or equal to the threshold and the second consists of patients above the chosen threshold. Only thresholds that split the cohort into groups larger than 10% of the cohort size were tested. We present an example schematic of this process to test the significance of lacunarity and fractal dimension in Fig. 1B. We present adjusted and unadjusted p-values within this work, and will clearly state when each is used.

Cox proportional hazard (CPH) models have been used predictors of survival in univariate and multivariate analyses against values that exist within all patients such as age at diagnosis and tumor radius. We used Pearson correlation coefficient tests to determine the significance and strength of correlations between variables. We used Welch’s t-tests to determine the significance between means of different groups.

### Ethical approval

All procedures performed in the studies involving human participants were in accordance with the ethical standards of the institutional and/or national research committee and with the 1964 Helsinki declaration and its later amendments or comparable ethical standards. Our de-identified data repository of patients with brain cancer includes retrospective data collected from medical records and prospective data collection. Research conduct on the data repository is approved by Mayo Clinic Institutional Review Board (IRB# 17-009688). Retrospective inclusion as well as informed consent was obtained for all prospectively enrolled participants in the repository as approved by Mayo Clinic Institutional Review Board (IRB# 17-009682).

## Results

### Whole cohort

#### Necrotic regions

We found that lacunarity significantly distinguished overall survival with the group of lower lacunarity values showing benefit, but this result did not hold while adjusting for multiple comparisons (unadjusted p = 0.012, adjusted p = 0.07541, n = 390). We also found that lacunarity significantly distinguished progression free survival. Lacunarity could still significantly distinguish PFS while accounting for multiple comparisons (adjusted p = 0.0051, n = 125).

#### Enhancement with necrotic regions

**Figure 2:**
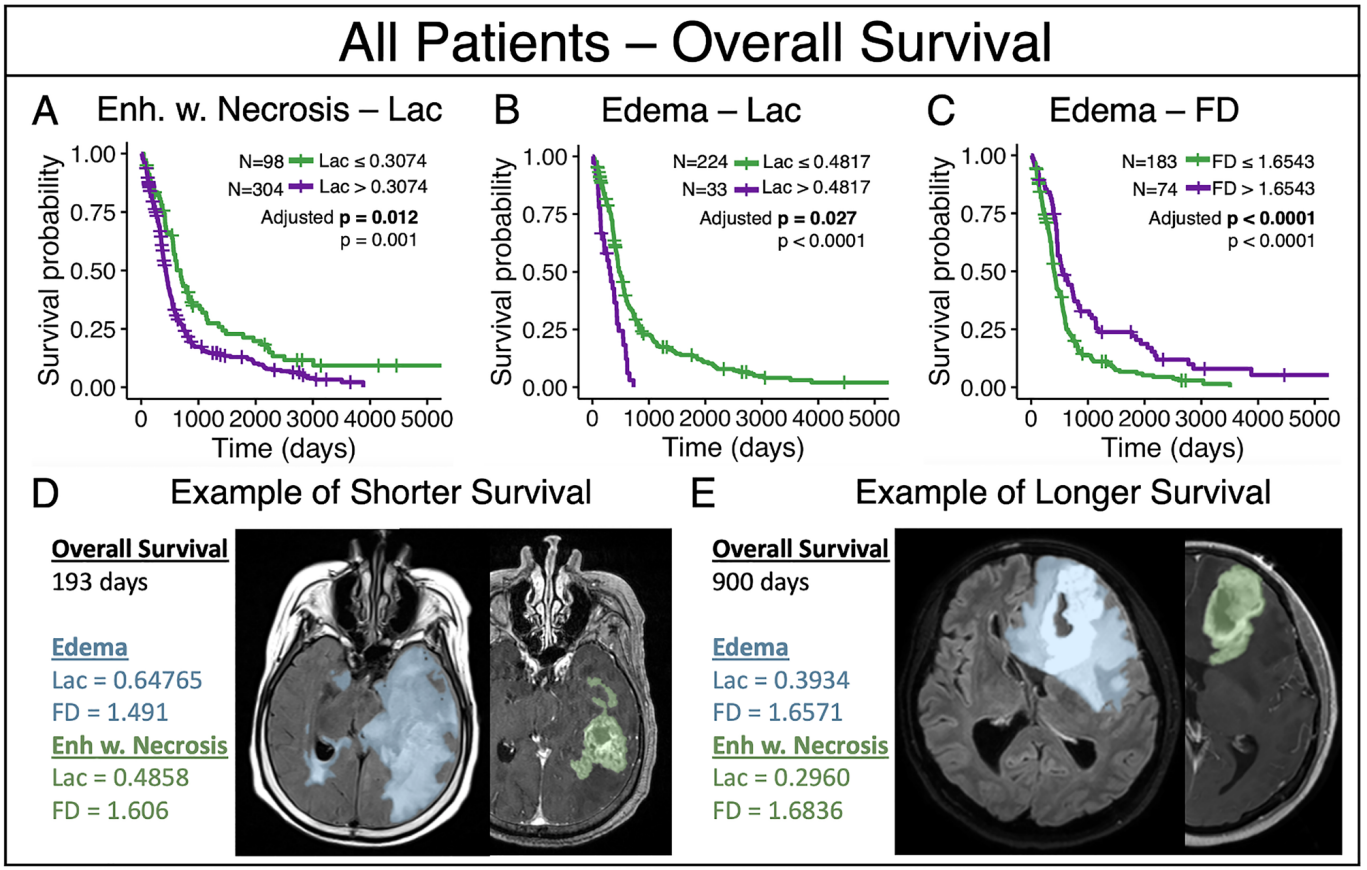
Results amongst all patients that remained significant with adjustment (**A**) The group of lower median lacunarity values of enhancement with necrosis (Lac ≤ 0.3074) were associated with prolonged overall survival. (**B**) The group of lower median lacunarity values of edema were associated with longer overall survival (Lac ≤ 0.4817). (**C**) The group of higher median fractal dimension values of edema were associated with longer overall survival (FD > 1.6543). (**D**) An example patient with lower overall survival of 193 days who consistently fell into the short overall survival groups of subpanels A-C. Example MRI slices shown with translucent segmentation overlays. (**E**) An example patient with longer overall survival of 900 days, who consistently fell into the longer overall survival groups of subpanels A-C. Example MRI slices shown with translucent segmentation overlays.

In T1Gd contrast with necrosis, we observe a lacunarity threshold of 0.3074 that significantly distinguishes groups for overall survival with the group of lower lacunarity values surviving longer (adjusted p = 0.012, n = 402), see Fig. 2A. We also saw that lacunarity distinguished PFS (unadjusted p = 0.015), but this did not remain significant when adjusting for multiple comparisons (adjusted p = 0.10).

#### Edema regions

In T2/FLAIR edema abnormalities, lower lacunarity (Lac ≤ 0.4817, adjusted p = 0.0269) and higher fractal dimension values (FD > 1.6543, adjusted p < 0.0001) were associated with significantly improved overall survival (n = 257), see Fig. 2B,C. We show examples of edema, and enhancement with necrosis for patients with a shorter and longer survival in Fig. 2D and E, respectively.

### Current standard of care

We implemented the same analyses within a sub-cohort of patients known to have been treated with the current standard of care, the so called Stupp Protocol which includes surgical resection to the maximal possible extent, combination radio-chemotherapy followed by adjuvant temozolomide chemotherapy^1,2^. Significant survival differences present in the whole cohort may not be reflected in this section due to a reduction in sample size leading to a reduction in statistical power.

#### Necrotic regions

**Figure 3:**
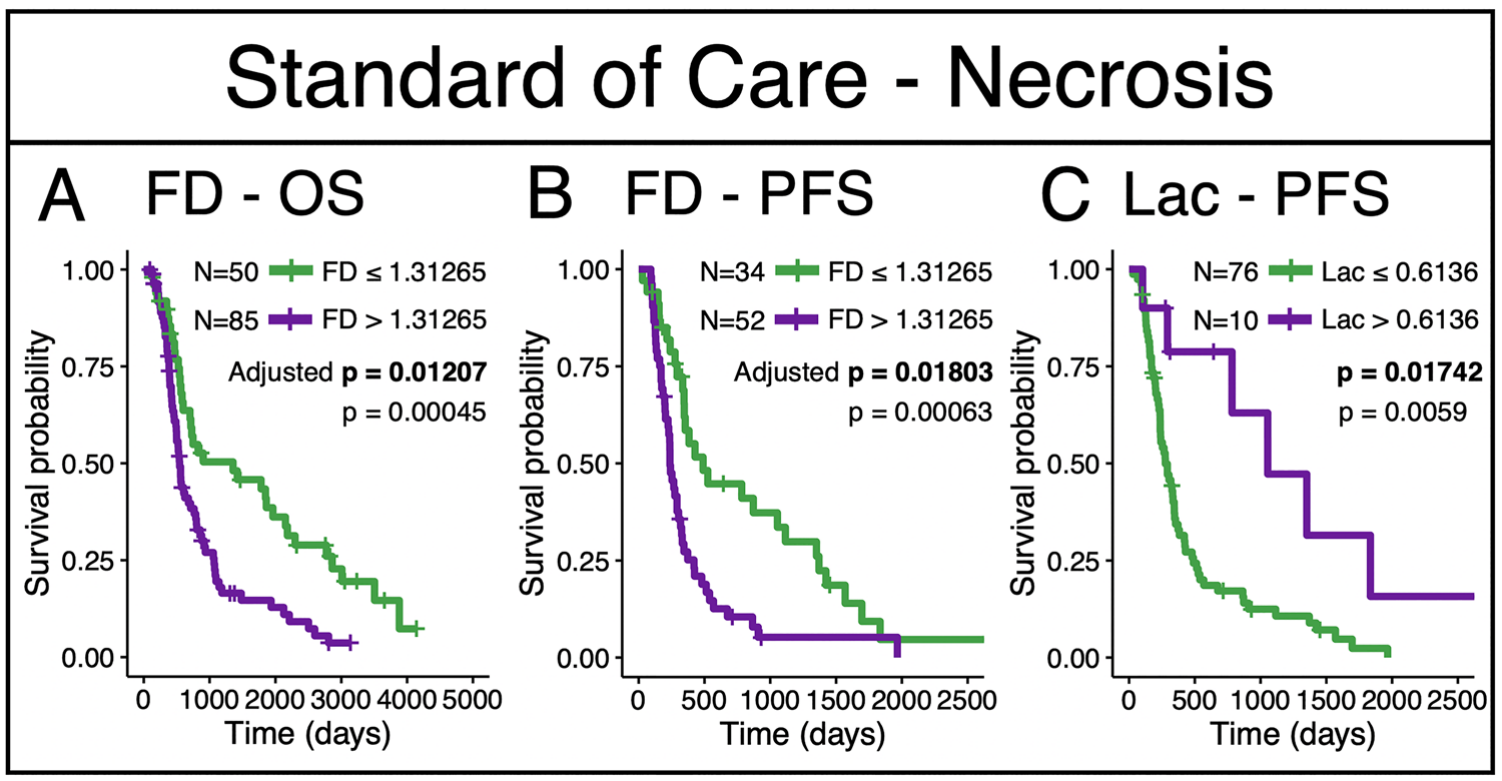
Fractal dimension and lacunarity cutoffs that significantly distinguish survival amongst patients confirmed to have received the current standard of care. (**A**) Fractal dimension of necrosis regions significantly distinguished OS. (**B**) Fractal dimension of necrosis regions significantly distinguished PFS. (**C**) Lacunarity significantly distinguished PFS. All three results remained significant with adjustment. Adjusted p values shown in bold.

In this subcohort of patients who received the current standard-of-care, we find that lacunarity and fractal dimension significantly distinguish overall survival and progression free survival. Lacunarity can distinguish progression-free survival while adjusting for multiple comparisons (adjusted p = 0.017, n = 86), with the same threshold chosen to separate the groups as was chosen in the whole cohort (Figs. 2A and 3). We also see that the fractal dimension of necrosis can significantly distinguish overall survival (adjusted p = 0.012, n = 135) and progression free survival (adjusted p = 0.018, n = 86) (Fig. 3) while accounting for multiple comparisons, with lower values conferring the survival benefit.

#### Enhancement with necrotic regions

Although no results held in this subcohort after adjusting for multiple comparisons, we did observe lacunarity thresholds that distinguished both overall and progression free survival with unadjusted significance (OS: p = 0.043, adjusted p = 0.31, PFS: p = 0.016, adjusted p = 0.091).

#### Edema regions

Although no results held when adjusting for multiple comparisons in this subcohort, we did observe similar optimal cutoffs to those found in the larger cohort for both lacunarity and fractal dimension that significantly distinguished overall survival without adjustment (lacunarity p = 0.019, FD p = 0.020), see Table 2. We also note that the optimal cutoff for fractal dimension distinguished progression free survival, but this result did not hold after adjustment for multiple comparisons (p = 0.013).

**Table 2:** Median lacunarity and fractal dimension (FD) tests that showed at least one significant cutoff that distinguishes survival.

	T1Gd				T2/FLAIR	
	Necrosis		Enhancement with necrosis		Edema	
	Lacunarity	FD	Lacunarity	FD	Lacunarity	FD
**All patients**						
OS	*0.27915*	n.s.	**0.3074**	n.s.	**0.4817**	**1.6543**
PFS	**0.6136**	*1.31265*	*0.3900*	n.s.	n.s.	n.s.
**Current SOC**						
OS	*0.28125*	**1.31265**	*0.3980*	n.s.	*0.456*	*1.6621*
PFS	**0.6136**	**1.31265**	*0.3923*	n.s.	n.s.	*1.5202*

We show both overall survival (OS) and progression free survival (PFS) with those that were significant (unadjusted p < 0.05) in italics. The results that remained significant while adjusting for multiple comparisons are shown in bold (adjusted p < 0.05). The numerical value in the cell represents the optimal threshold of analyses that reached a level of significance. Results that did not show significance are indicated by "n.s" to represent "not significant".

We present a summary of the cutoffs that were found to be significant in either an unadjusted log-rank test or with adjustment (as described in Lausen and Schumacher^33^) in Table 2. We also present sex-specific breakdowns of these analyses in Supplement 1.

### Univariate and multivariate Cox proportional hazard models

**Figure 4:**
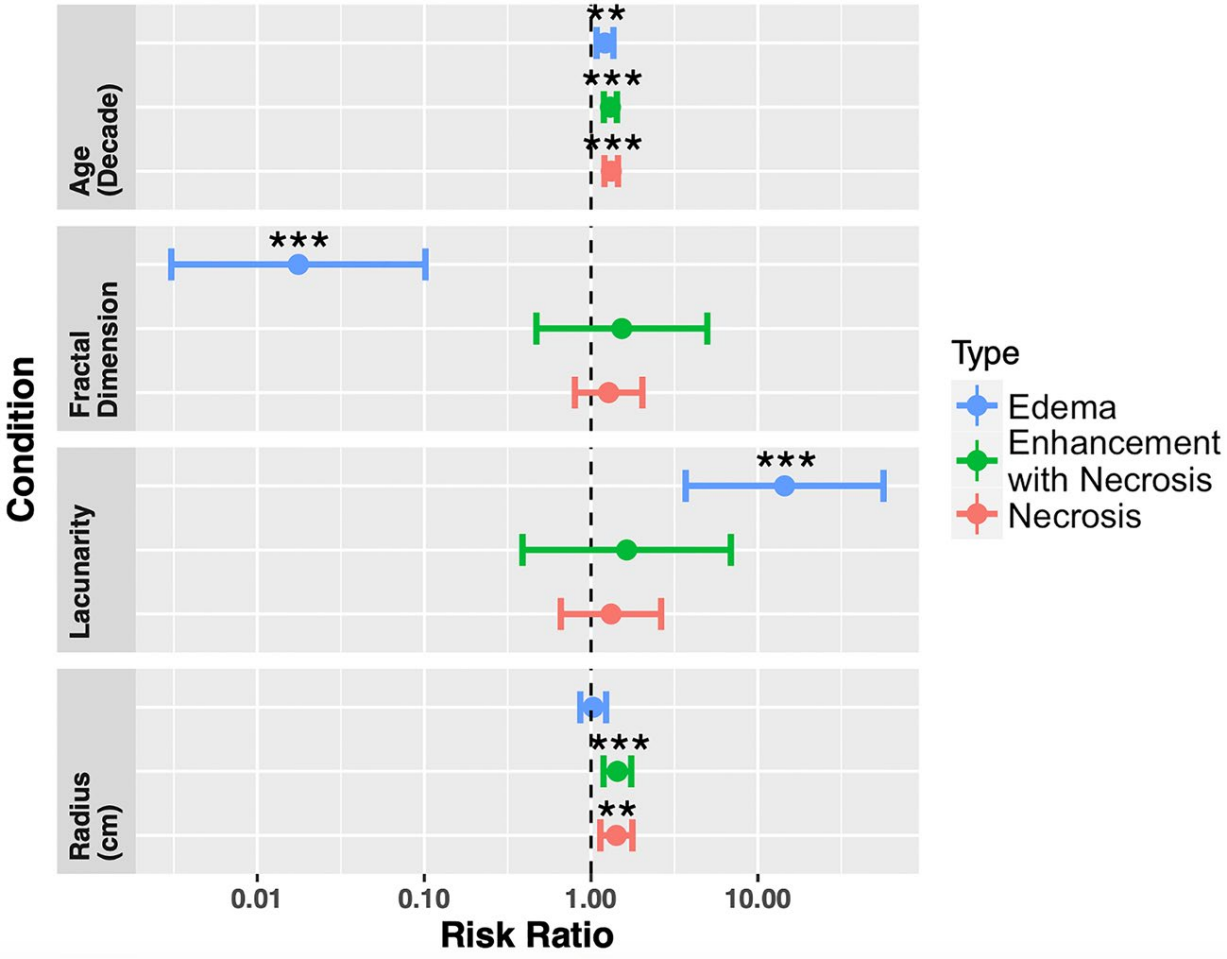
Univariate Cox proportional hazard models for age at diagnosis, fractal dimension, lacunarity and tumor radius, across all three regions (necrosis n = 390, enhancement with necrosis n = 402, edema n = 257). Both fractal dimension and lacunarity of edema-related abnormalities are significant prognostic indicators of overall survival. Age at diagnosis was significant for all three regions, while radius was significant for both necrosis and enhancement with necrosis. Values of these tests can be found in Supplement 4.

**Figure 5:**
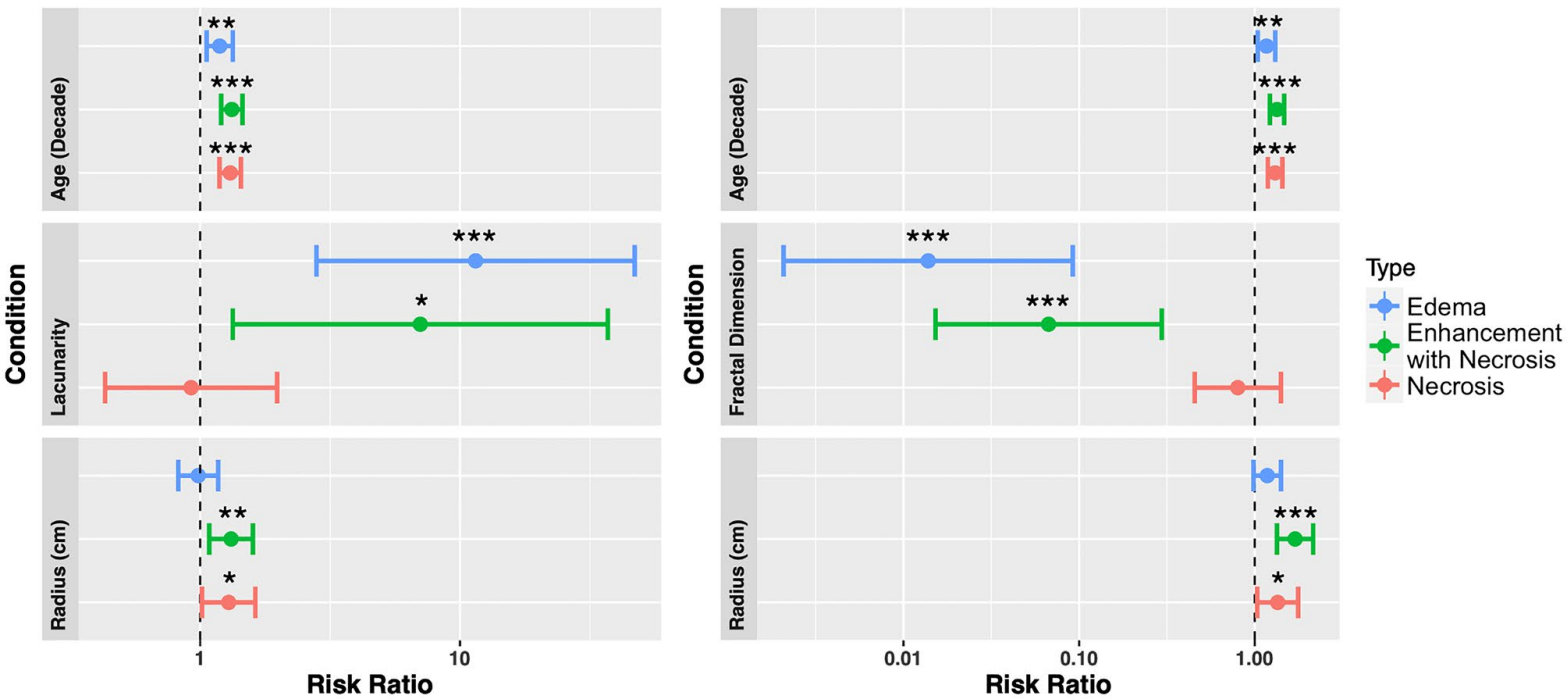
(Left) Multivariate CPH models of lacunarity, age at diagnosis, and abnormality radius for necrosis (n = 390), enhancement with necrosis (n = 402), and edema regions (n = 257). Lacunarity of both edema and enhancement with necrosis were significant predictors of overall survival in their respective models (p = 0.0007 and p = 0.042, respectively). Age at diagnosis, as expected, was consistently significant for survival. Radius was a significant predictor for regions of necrosis and enhancement with necrosis (p = 0.036). (Right) Corresponding Cox proportional hazard model of fractal dimension, age at diagnosis, and abnormality radii for necrosis, enhancement with necrosis, and edematous regions. Fractal dimension values of both edema and enhancement with necrosis were significant predictors of overall survival in their respective CPH models (p < 0.0001 and p = 0.0003, respectively). Age at diagnosis was consistently significant across all CPH models, while only enhancement with necrosis, and necrosis radii were significant (p = 0.0018 and p = 0.028, respectively). For a complete table including confidence intervals and significance values, see Supplement 4.

We implemented univariate and multivariate Cox proportional hazard (CPH) models against overall survival and progression free survival. For overall survival, we present the univariate CPH models for the necrosis, T1Gd, and T2/FLAIR regions for age at diagnosis, fractal dimension, lacunarity, and tumor radius at presentation in Fig. 4 (across the entire cohort). Multivariate CPH models assessing the relationship between overall survival and lacunarity, age at diagnosis, and tumor radius are on the left panel of Fig. 5 and multivariate analyses of fractal dimension, age at diagnosis and tumor radius are on the right panel of Fig. 5. We find that lacunarity and fractal dimension values of T2/FLAIR regions are the most commonly significant in univariate and multivariate analyses. The values found in Figs. 4 and 5 are presented in tables in Supplement 4. We chose to run two separate multivariate CPH models, one with fractal dimension and another with lacunarity, to test their independent ability as prognostic indicators against other factors. We present the equivalent results amongst patients known to have received the current SOC in Supplement 5, which also show significance of both fractal dimension and lacunarity of T2/FLAIR for overall survival. We found that in a multivariate CPH analysis for progression-free survival of lacunarity of necrosis, necrosis radius, and age at diagnosis (across the entire cohort), that lacunarity and radius were both significant. No other variables were found to be significant for progression-free survival in either univariate or multivariate CPH analyses for any regions. We present plots of these results in Supplement 6. For a subset of patients with available extent of resection (necrosis n=263, enhancement with necrosis n=274, edema n=155), we present the analogous multivariate CPH analyses in Supplement 7. All significant necrosis and edema results pertaining to morphology persist in this reduced cohort setting, while enhancement with necrosis results did not.

### Correlations with other variables

We see significant negative correlations between lacunarity and fractal dimension within all of the imaging abnormalities tested (necrosis R = − 0.55 p < 0.0001, enhancement with necrosis R = − 0.45, p < 0.0001, edema R = − 0.55, p < 0.0001). Except for lacunarity of enhancement with necrosis (p = 0.08), both lacunarity and fractal dimension are consistently significantly positively correlated with their corresponding volumes (all tests p < 0.001).

Within the cohort of patients for which we have all three regions available (n = 250), we observe significant positive correlations between both lacunarity and fractal dimension values of necrotic regions and enhancement with necrosis (both tests p < 0.0001 Pearson). Significant positive correlations are also present between these metrics calculated on enhancement with necrosis and edema regions (lacunarity p = 0.009 and FD p = 0.018). We did not observe significant correlations between these metrics calculated on necrotic regions and edema regions (lacunarity p = 0.185 and FD p = 0.339).

**Figure 6:**
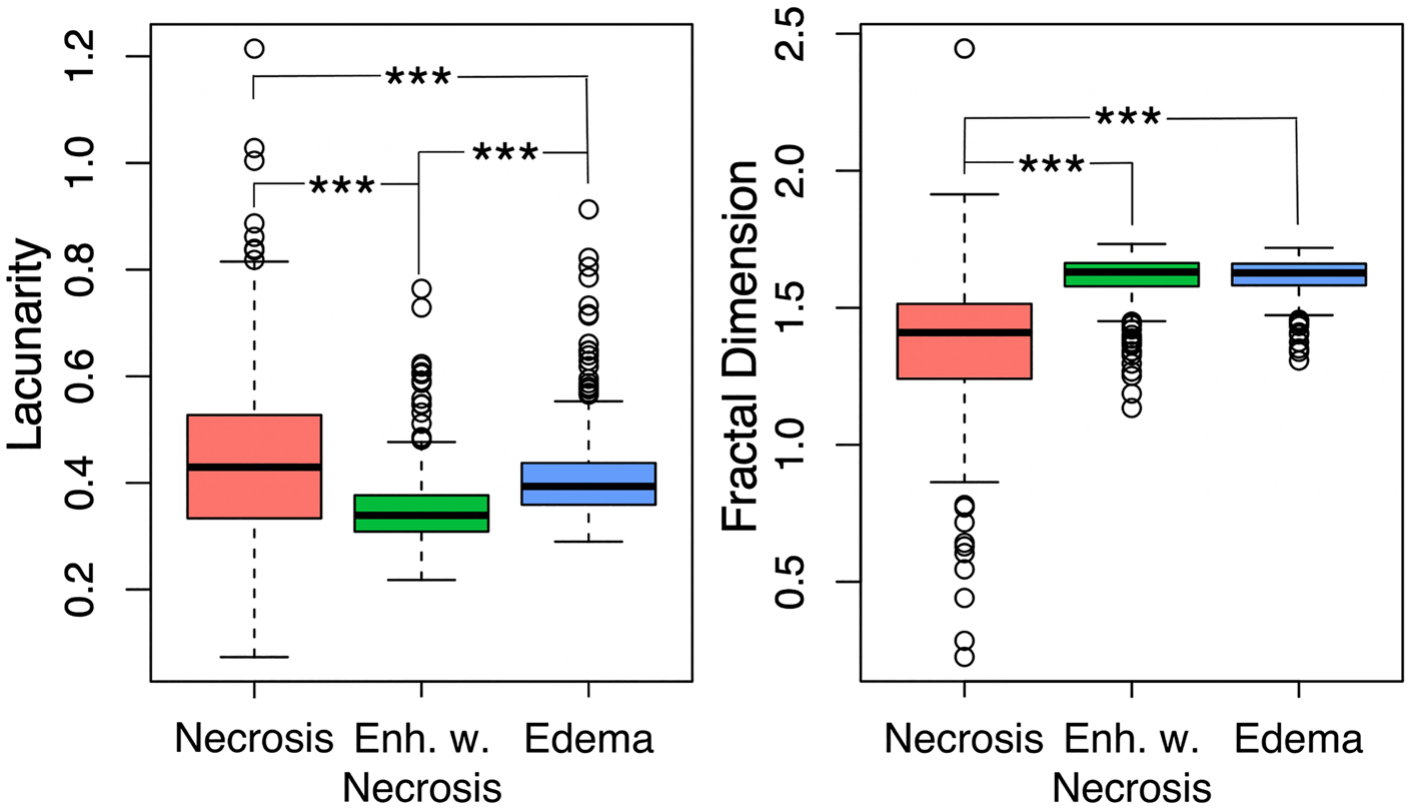
Significant differences in lacunarity and fractal dimension between regions of interest. (Left) We note significant differences in lacunarity values across all three regions of interest, with necrosis the highest, followed by edema, followed by enhancement with necrosis (all comparisons p < 0.001). (Right) Fractal dimension values are significantly higher in edema and enhancement with necrosis when compared with necrosis (p < 0.001), but we did not observe significant differences in fractal dimension between edema and enhancement with necrosis (p = 0.49).

We see significantly lower fractal dimension values in necrosis-related abnormalities compared with their counterparts with enhancement (p < 0.0001 t-test) and edema (p < 0.001 t-test). No significant difference is observed in the fractal dimension between enhancement with necrosis and edema (p = 0.49, t-test). We see significance between all three regions in lacunarity. Lacunarity is significantly higher in edema than enhancement with necrosis (p < 0.001, t-test), and necrosis is significantly higher than regions of edema (p < 0.001, t-test). Figure 6 shows boxplots of these values with their significant relationships highlighted.

We found that lacunarity was significantly associated with image resolution across all three abnormalities tested, while fractal dimension was associated with both enhancement with necrosis, and regions of edema. We present details of this analysis in Supplement 8. Although we see these significant relationships, lacunarity and fractal dimension results of analyses presented in Fig. 5 were unaffected by the inclusion of image resolution (Supplement 8).

**Figure 7:**
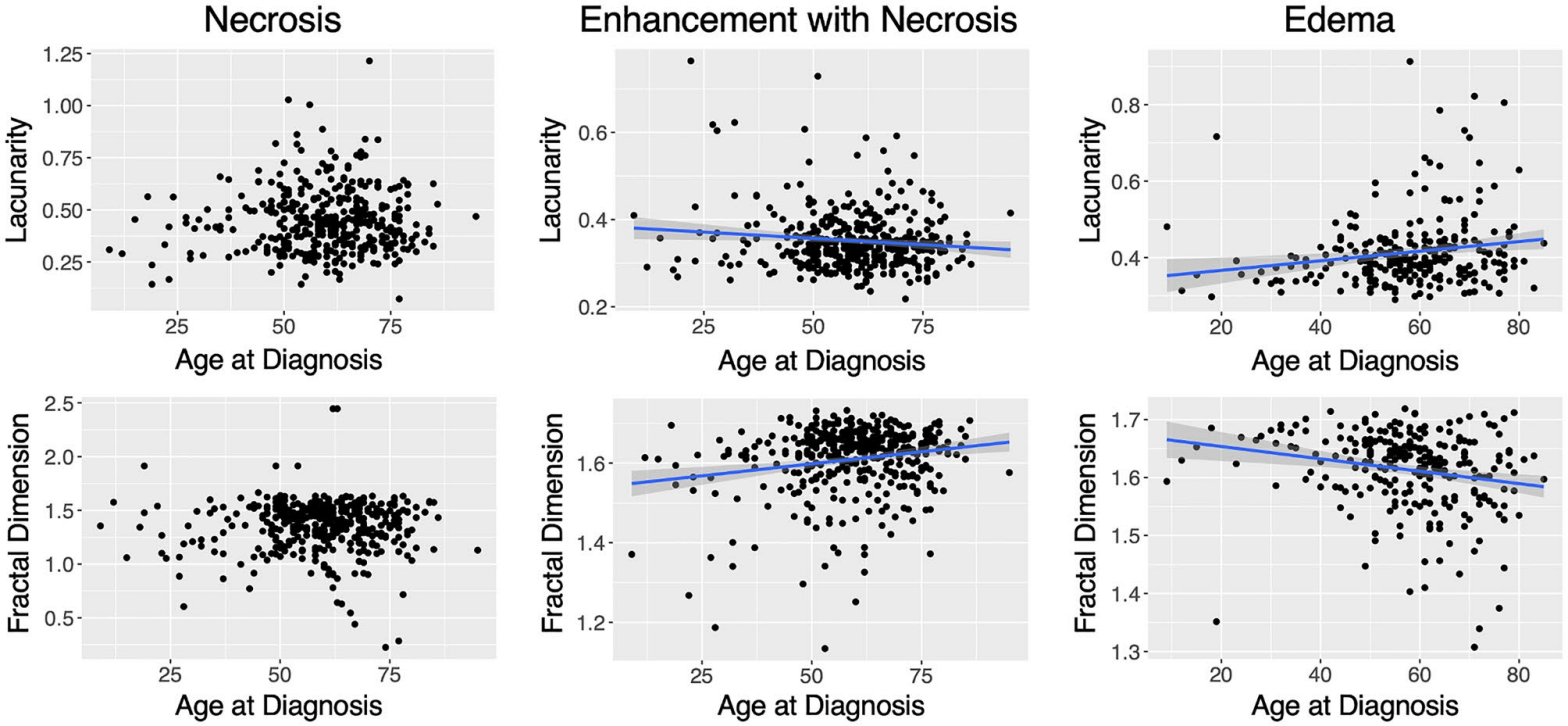
Significant relationships between morphology and age reversed between enhancement with necrosis and edematous regions. (Top row) We see a weak significantly negative correlation between lacunarity and age at diagnosis in enhancement with necrosis ROIs (p = 0.0217, R = − 0.11) and a weak significantly positive correlation within edema ROIs (p = 0.0035, R = 0.18). (Bottom row) We see weak significant relationships between fractal dimension and age at diagnosis within enhancement with necrosis (p < 0.001, R = 0.19) and edema ROIs (p < 0.001, R = − 0.21), respectively. Fractal dimension of enhancement with necrosis is positively correlated with age at diagnosis whereas the fractal dimension of edema ROIs has a negative correlation. Trend lines are shown for significant correlations.

We note significant correlations of both morphological metrics with age at diagnosis. We observe a weak negative significant correlation between lacunarity and age at diagnosis in enhancement with necrosis (p = 0.0217, R = -0.11 Pearson) but a positive significant correlation in edema regions (p = 0.0035, R = 0.18, Pearson). In contrast to this, we note a significant positive correlation between fractal dimension and age at diagnosis in enhancement with necrosis (p < 0.001, R = 0.19, Pearson) and a significant negative correlation in edema regions (p < 0.001, R = -0.21, Pearson). We do not observe significant correlations within necrotic regions of lacunarity or fractal dimension with age at diagnosis. These results are shown in Fig. 7.

## Discussion

Although clinical care teams holistically consider multiple factors to determine the best course of action for each patient with GBM, they are somewhat limited in the computational tools and prognostic indicators that are readily available to them. The limited opportunities for tissue collection leads to a clinical reliance on imaging to make these decisions and an opportunity to maximize the utility of this information through prognostic imaging-derived biomarkers. Through a multi-institutional retrospective cohort, we present highly reliable (Supplement 3) imaging metrics that suggest the shape of GBM has prognostic value at presentation. In contrast to the previous publication on this topic^7^, we see some opposing relationships with these morphological metrics and patient survival for necrotic regions, in that our results generally show a low fractal dimension (less circular, patchier) in necrotic regions is better for patient survival. To compare more closely with the previous publication^7^, we also ran survival analyses for the mean lacunarity and fractal dimension values on necrotic regions. These metrics did not significantly distinguish overall survival and progression free survival in as many instances as the median (Supplement 2).

Within necrotic regions, we see more significant relationships between these morphological metrics and patient survival within those who received the current standard of care (Fig. 3). In this setting, fractal dimension can distinguish overall survival and progression free survival (Fig. 3A,B), and lacunarity can distinguish progression free survival while adjusting for multiple comparisons (Fig. 3C). Lacunarity also significantly distinguished overall survival, but this result did not hold when adjusting for multiple comparisons. These results suggest that less connected and more heterogeneous necrosis indicates longer PFS and OS for patients with GBM receiving the current standard of care. Patchier and less-connected necrosis may indicate that the necrosis is less well established.

The lacunarity of pretreatment lesions present on T2 MRI has previously been shown to distinguish glioma grade^21^. We have extended on this result to suggest that the shape of these regions also contain information on patient survival within GBM. Notably, in our cohort, we observe a benefit to OS of lower edema lacunarity values and high edema fractal dimension values, both within our optimal threshold analysis and as continuous variables in univariate and multivariate CPH analyses. These results suggest that patchier and more heterogeneous edematous regions provide a worse prognosis. Within patients receiving the current standard of care, we also see that lacunarity and fractal dimension of edema-related abnormalities act as independent prognostic variables against age at diagnosis and the T2/FLAIR abnormality radius. These results suggest meaningful clinical insights may come from quantifying the morphological appearance of GBMs. Future work may connect these imaging metrics with biological drivers of clinical aggressiveness through molecular and histological analysis of tissue specimens.

We tested regional volumes as they relate to outcome, but these did not subsume results of lacunarity and fractal dimension; this was particularly clear for edema. Rather unexpectedly, we also note that lacunarity and fractal dimension weakly correlate with patient age for enhancement with necrosis and edematous regions. Machine learning has been used to reliably predict patient age from brain MRI of healthy adults^34^, but to our knowledge no work has noted relationships between patient age and brain tumor shape. Further, the connection between these morphological metrics and age also suggests that tumors that develop in aged brains may have different biological aggressiveness. Notably, the fact that age and these morphological metrics retain independent prognostic value suggests that although increasingly aggressive appearing tumors are increasingly common with age, there are additional insights to be found in the morphology.

It is important to note that a lack of statistical significance in survival analyses does not necessarily mean a lack of signal. In stratifying our patient cohorts, our statistical power to observe potential differences decreases. We chose to present optimal thresholds that did not remain significant while adjusting for multiple comparisons to show that we do see some signal with these morphological metrics in most cases. We hope that in the future these optimal thresholds will be tested in an independent patient cohort to validate the results presented here.

We computed 2D lacunarity and fractal dimension values of each MRI slice (and averaged these for each patient) rather than computing 3D values. Although of course tumors develop in 3D space, the resolution of MRI is typically lower between slices than within slices, which limits our ability to ascertain accurate lacunarity and fractal dimension values in 3D space.

There has been some recent research showing that GBM location within the brain can impact outcome^35,36^. A future direction may explore lacunarity and fractal dimension against the tumor location, to determine whether location compliments or drives prognostic signals of lacunarity and fractal dimension that we have observed here. Further work may also explore the dynamics of these morphological markers throughout treatment and tumor progression.

Throughout this work, we have found relationships between the shape of segmented tumor regions and survival metrics. We found lacunarity and fractal dimension thresholds that significantly distinguish patient overall survival and progression-free survival in our cohort, and showed that these act as continuous predictors of survival in some cases. These results warrant further investigation into the biological and genetic drivers behind the morphological presentation of GBM in a pretreatment setting.

## Supplementary Information

Supplementary Information.

## Abbreviations

GBM: Glioblastoma
FD: Fractal dimension
MRI: Magnetic resonance imaging
FLAIR: Fluid-attenuated inversion recovery
T1Gd: Gadolinium-enhanced T1-weighted
OS: Overall survival
PFS: Progression free survival
CPH: Cox proportional hazard
