# Supplementary material for: Shape matters: morphological metrics of glioblastoma imaging abnormalities as biomarkers of prognosis
Source: Sci Rep. 2021 Dec 1;11:23202. doi: 10.1038/s41598-021-02495-6 (PMC8636508; doi:10.1038/s41598-021-02495-6)
Supplement: Supplementary file 1 — Supplementary Information. [file 41598_2021_2495_MOESM1_ESM.pdf]

Supplemental Data - Shape Matters: Morphological Metrics of Glioblastoma Imaging Abnormalities as Biomarkers of Prognosis

Supplement 1: Sex-specific Results

All results presented in the previous subsections were carried out on a sex-specific basis.

		T1Gd MRI						T2/FLAIR MRI		
		Necrosis			Enhancement with necrosis			Edema		
		All	Male	Female	All	Male	Female	All	Male	Female
Everyone	OS	390	246	144	402	253	149	257	164	93
	PFS	125	84	41	130	87	43	78	50	28
Current SOC	OS	135	88	47	142	92	50	92	56	36
	PFS	86	58	28	89	59	30	56	33	23

Cohort numbers for patients with known overall survival (OS) and progression free survival (PFS). This table shows the patients with known OS and PFS, including the subsets known to have received the current standard of care (SOC). We also present how these cohorts are split by patient sex. The discrepancy between patients with necrosis ROIs and T1Gd enhancing is due to 10 patients with negligible necrosis that did not meet our criteria to be included in this retrospective study.

		T1Gd MRI						T2/FLAIR MRI		
		Necrosis			Enhancement with necrosis			Edema		
		All	Male	Female	All	Male	Female	All	Male	Female
Everyone	OS	0.27915	0.33525	0.2919	0.3074	0.3074	0.30305	0.4817	0.4817	0.4705
	PFS	0.6136	0.6136		0.39		0.3893			

Current SOC	OS	0.28125			0.398			0.456		0.37355
	PFS	0.6136		0.6011	0.3923		0.3816	0.34695		

Median lacunarity tests that showed at least one significant cutoff that distinguishes survival. We show both overall survival (OS) and progression free survival (PFS) with those that were significant in light gray. The results that remained significant while adjusting for multiple comparisons are shown in dark gray.

		T1Gd MRI						T2/FLAIR MRI		
		Necrosis			Enhancement with necrosis			Edema		
		All	Male	Female	All	Male	Female	All	Male	Female
Everyone	OS			1.26725			1.565	1.6543	1.64625	1.6541
	PFS	1.31265	1.31265					1.62515		1.62305
Current SOC	OS	1.31265	1.31265	1.26725		1.675	1.565	1.6621	1.51575	1.66355
	PFS	1.31265	1.31265					1.62515		

Median fractal dimension tests that showed at least one significant cutoff that distinguishes survival. We show both overall survival (OS) and progression free survival (PFS) with those that were significant in light gray. The results that remained significant while adjusting for multiple comparisons are shown in dark gray.

Supplement 2: Testing Mean Lacunarity and Mean Fractal Dimension

		T1Gd: Necrosis			T1Gd: Enhancement with Necrosis			T2/FLAIR: Edema		
		All	Male	Female	All	Male	Female	All	Male	Female
Everyone	OS	0.3405833	0.3405833		0.3051281	0.3039867	0.3016833	0.4915923	0.4504357	0.4820333
	PFS	0.606835						0.3495091	0.3495091	0.3699375
Current SOC	OS				0.3069367	0.3069367		0.4820333	0.4915923	
	PFS	0.6120233				0.2833917	0.3824581	0.3540625	0.3540625	

Mean lacunarity tests that showed at least one significant cutoff that distinguishes survival. We show both overall survival (OS) and progression free survival (PFS) with those that were significant in light gray. The results that remained significant while adjusting for multiple comparisons are shown in dark gray.

		T1Gd: Necrosis			T1Gd: Enhancement with Necrosis			T2/FLAIR: Edema		
		All	Male	Female	All	Male	Female	All	Male	Female
Everyone	OS			1.348725			1.547462	1.624938	1.624938	
	PFS	1.251472	1.238051					1.622186		
Current SOC	OS	1.2525					1.5958	1.650638		1.6441
	PFS	1.251472	1.238051	1.251472			1.587316	1.608108		

Mean fractal dimension tests that showed at least one significant cutoff that distinguishes survival. We show both overall survival (OS) and progression free survival (PFS) with those that were significant in light gray. The results that remained significant while adjusting for multiple comparisons are shown in dark gray.

Supplement 3: Intraclass Coefficients of Lacunarity and Fractal Dimension Values

On a subset of images with multiple segmentations available (most had two, some with three or four), we tested the intraclass coefficients (ICCs) of lacunarity and fractal dimension across the three imaging abnormalities presented in the main text. This was carried out using the two-way case with multiple raters in the irrNA package in R (1).

We present the table of ICC values and 95% confidence intervals. ICC values range from 0 to 1, with 1 being perfect agreement. Significance for each test was p<1e-15 (exact values below resolution of R package). We see excellent agreement across all abnormality regions, with fractal dimension of necrosis presenting the lowest ICC.

	T1Gd MRI		T2/FLAIR MRI
	Necrosis (N=201)	Enhancement with necrosis (N=211)	Edema (N=136)
Lacunarity	0.937<br>[0.918, 0.952]	0.898<br>[0.867, 0.922]	0.942<br>[0.920, 0.958]
Fractal Dimension	0.898<br>[0.866, 0.922]	0.917<br>[0.892, 0.936]	0.916<br>[0.882, 0.940]

Supplement 4: Values Presented in Cox Proportional Hazard Plots

**Necrosis Variables (p value, HR, 95% CI)**	
**Univariate CPH Outcomes**	
Lacunarity	0.435, 1.317, [0.6590, 2.6332]
Fractal Dimension	0.307, 1.2744, [0.7999, 2.0303]
Age (decades)	**<0.0001, 1.3207, [1.2033, 1.4497]**
Radius (cm)	**0.00194, 1.4183, [1.1371, 1.7690]**
**Multivariate CPH Outcomes**	
Lacunarity	0.8351, 0.9223, [0.4305, 1.9757]
Age (decades)	**<0.0001, 1.3048678, [1.1867259 1.434771]**
Radius (cm)	**0.0364, 1.2872644, [1.0161378 1.630733]**
Fractal Dimension	0.441, 0.80070990, [0.454778154 1.40977825]
Age (decades)	**<0.0001, 1.30563149, [1.187578119 1.43542017]**
Radius (cm)	**0.028, 1.35074734, [1.032977624 1.76627096]**

**Enh. With Necrosis Variables (p value, HR, 95% CI)**	
**Univariate CPH Outcomes**	
Lacunarity	0.503, 1.63494814, [0.388452428 6.8812942]
Fractal Dimension	0.48, 1.52922637, [0.470292430 4.9725089]
Age (decades)	**<0.0001, 1.30594705, [1.193211710 1.4293337]**
Radius (cm)	**0.0002, 1.43847096, [1.187303246 1.7427719]**
**Multivariate CPH Outcomes**	

Lacunarity	**0.02143, 7.0339701, [1.3345471 37.073803]**
Age (decades)	**<0.0001, 1.3229636, [1.2037698 1.453960]**
Radius (cm)	**0.00552, 1.3147489, [1.0836820 1.595085]**
Fractal Dimension	**0.000356, 0.06702770, [0.015206290 0.29545094]**
Age (decades)	**<0.0001, 1.33902267, [1.219116419 1.47072230]**
Radius (cm)	**<0.0001, 1.69721089, [1.338093669 2.15270789]**

**Edema Variables (p value, HR, 95% CI)**	
**Univariate CPH Outcomes**	
Lacunarity	**0.00013, 14.44702788, [3.689517402 56.5701668]**
Fractal Dimension	**<0.0001, 0.01760181, [0.003048264 0.1016394]**
Age (decades)	**0.0011, 1.21317106, [1.080767757 1.3617949]**
Radius (cm)	0.746, 1.02989506, [0.861678973 1.2309501]
**Multivariate CPH Outcomes**	
Lacunarity	**0.000693, 11.4824765, [2.8026852 47.043195]**
Age (decades)	**0.003493, 1.1885148, [1.0584508 1.334561]**
Radius (cm)	0.844741, 0.9824919, [0.8232941 1.172473]
Fractal Dimension	**<0.0001, 0.01383143 0.002077994 0.09206398**
Age (decades)	**0.00873, 1.16620359 1.039598702 1.30822674**
Radius (cm)	0.07375, 1.17864640 0.984331381 1.41132077

Supplement 5: Current Standard of Care Cox Proportional Hazard Plots

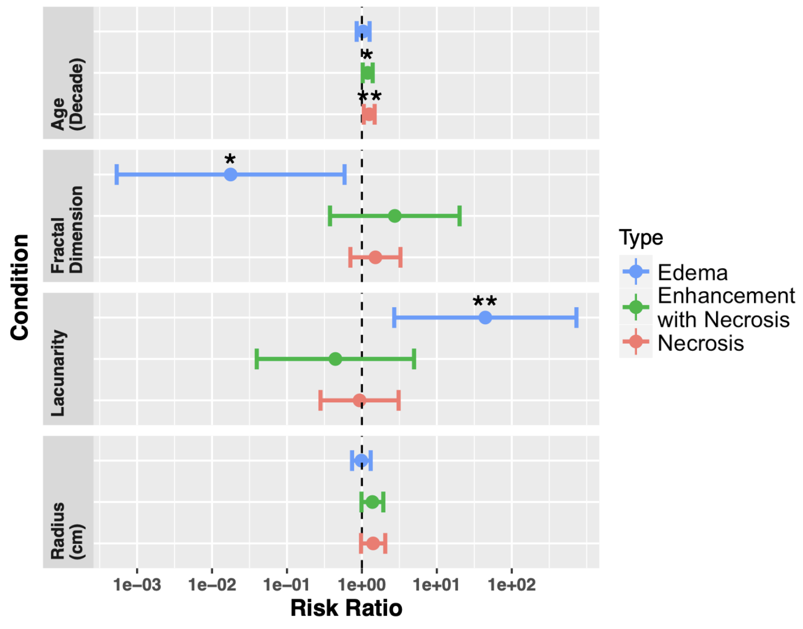

Current SOC - Univariate Analysis - Overall Survival

Univariate Cox proportional hazard model for overall survival of patients known to have received the current SOC. Patient counts are 135, 142, 92 for necrosis, enhancement with necrosis and edema analyses, respectively. Both fractal dimension and lacunarity are significant for edematous regions. Age at diagnosis was significant only for necrosis and enhancement with necrosis. The lack of signal for age at diagnosis for edema could be owing to the lower sample size.

Current SOC - Multivariate Analysis - Overall Survival

Multivariate Cox proportional hazard model for overall survival of patients known to have received the current standard of care. Separate analyses for necrosis (n=135), enhancement with necrosis (n=142) and edema (n=92) are all presented here. **(Left)** Lacunarity of edema showed significant influence for overall survival. The only other variable to show significance was age at diagnosis within the necrosis analysis. **(Right)** Fractal dimension was significant only for edematous regions, with age at diagnosis significant in analyses of necrosis and enhancement with necrosis.

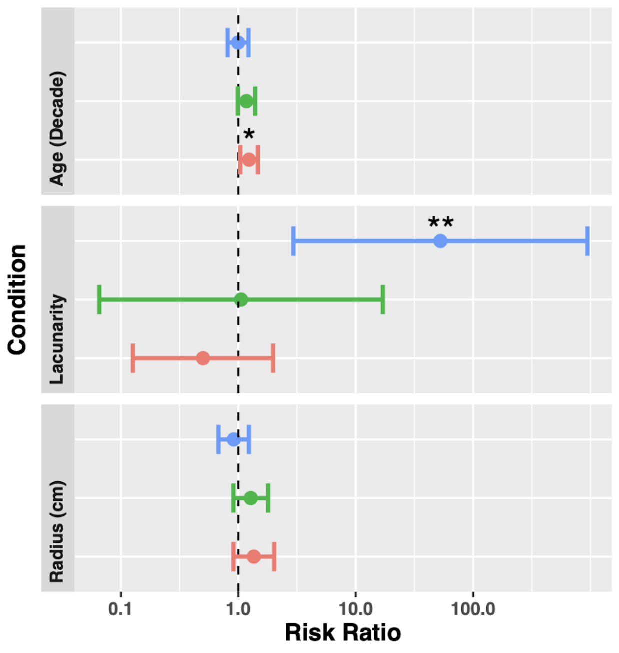

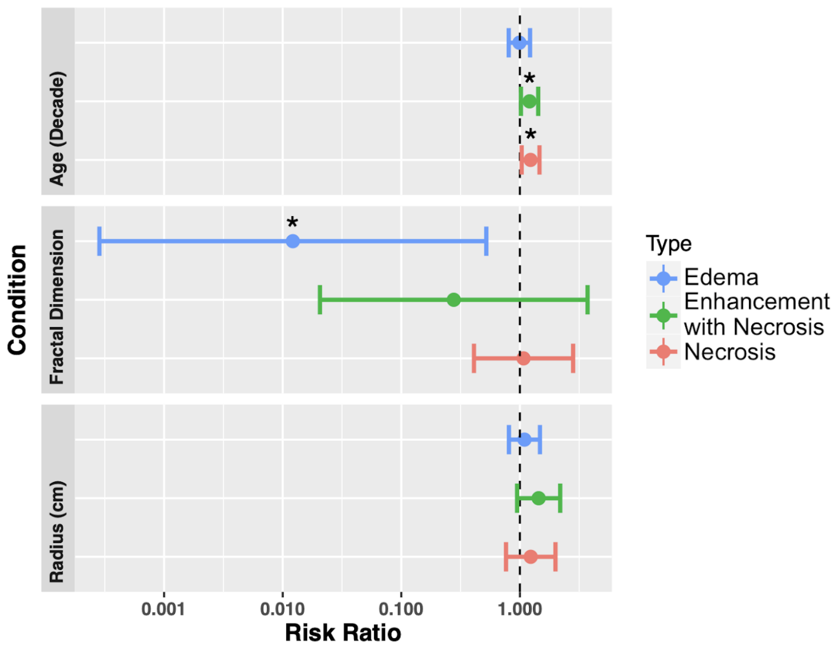

Supplement 6: Progression-Free-Survival Cox Proportional Hazard Plots

Univariate CPH for Progression-Free Survival

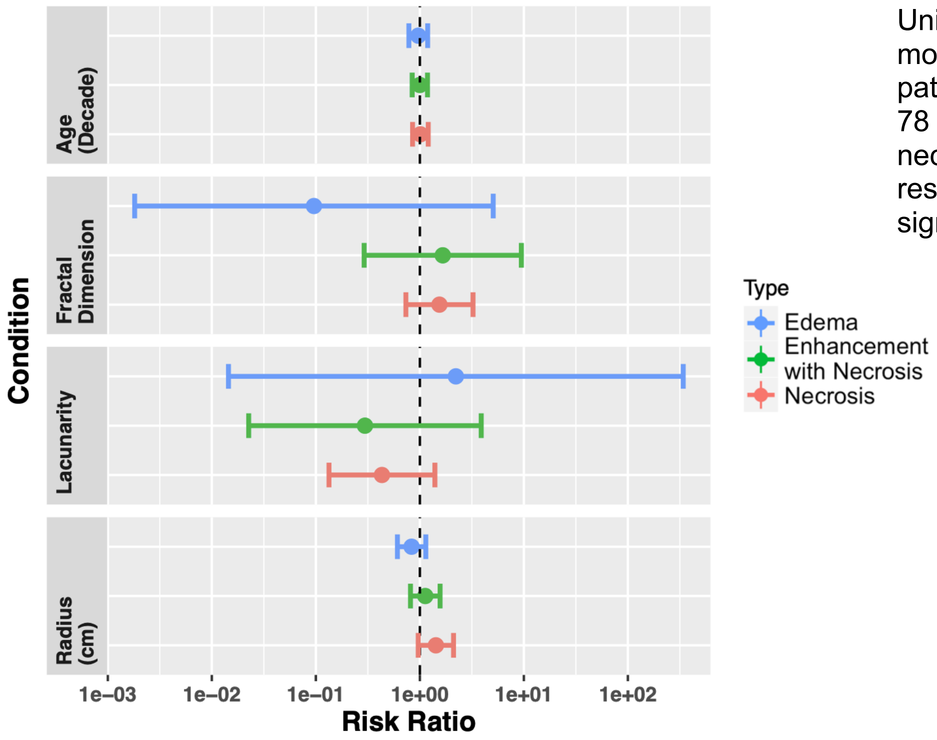

Univariate Cox proportional hazard model for progression-free survival of all patients. Patient counts are 125, 130 and 78 for necrosis, enhancement with necrosis and edema analyses, respectively. No variables showed significance in any of these analyses.

Multivariate CPH for Progression-Free Survival

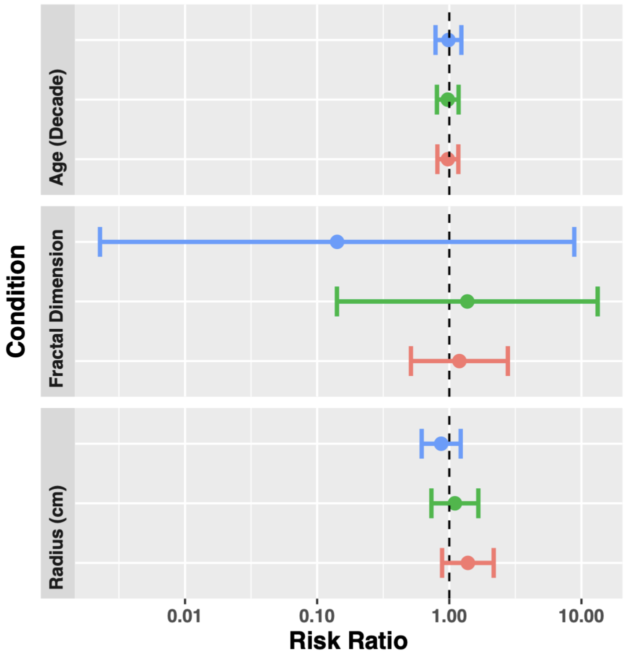

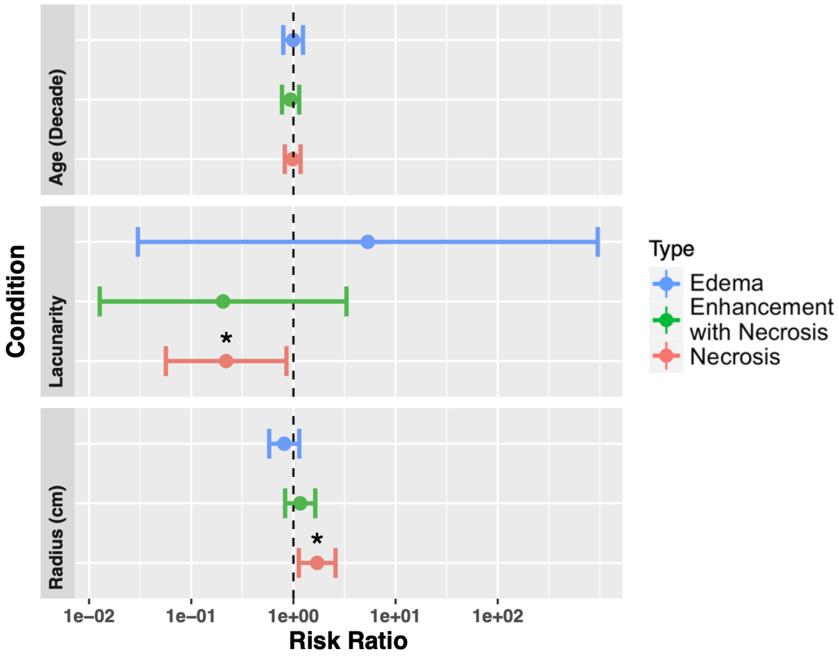

Multivariate Cox proportional hazard model for progression-free survival. Separate analyses for necrosis (n=125), enhancement with necrosis (n=130) and edema (n=78) are all presented here. **(Left)** No significance was found for any variables. **(Right)** Lacunarity of enhancement with necrosis showed significant influence for overall survival. The only other variable to show significance was necrosis radius.

Supplement 7: Including Extent of Resection in Cox Proportional Hazard Plots

Multivariate CPH for Overall Survival

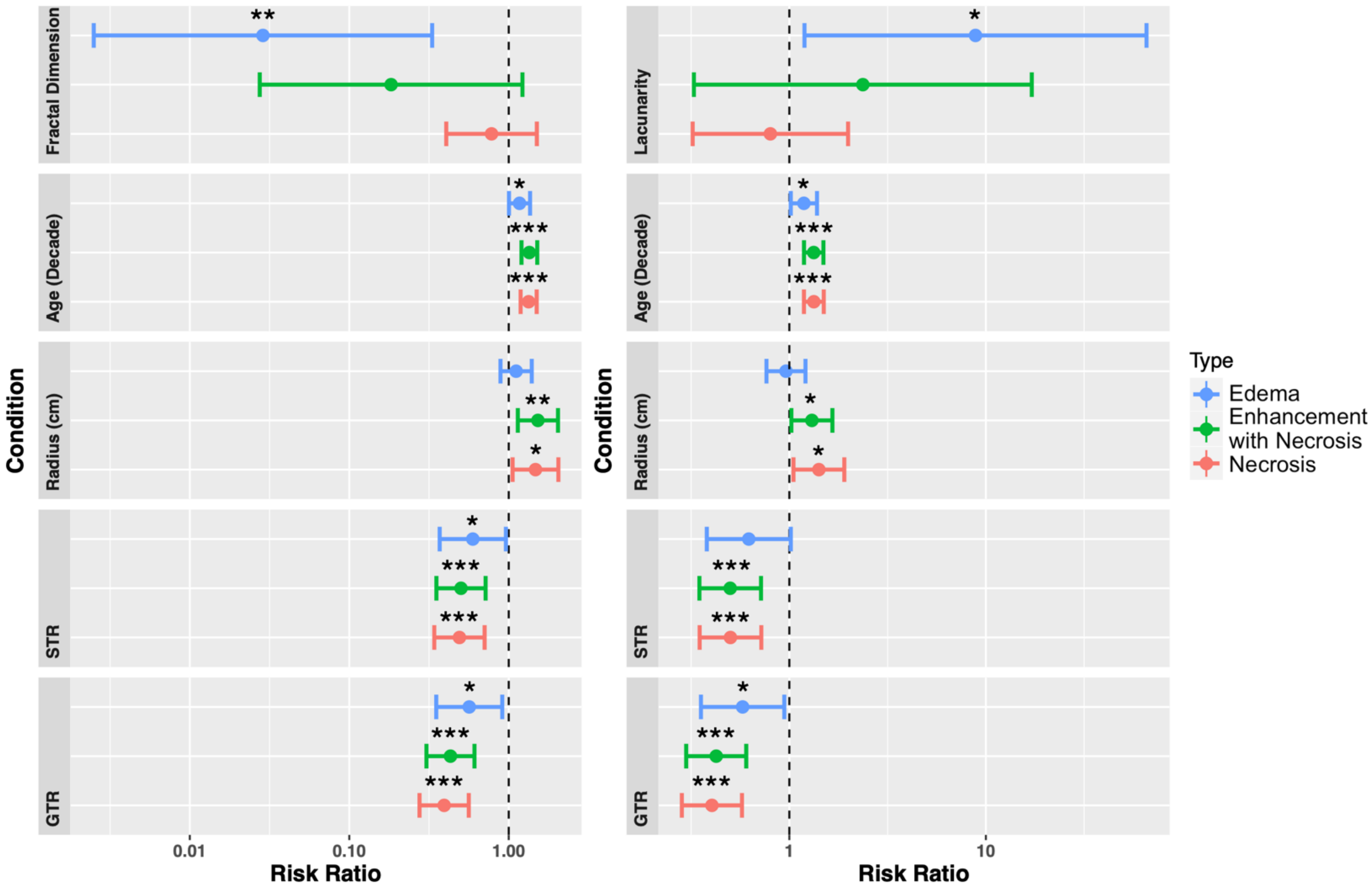

Multivariate Cox proportional hazard model for overall survival. Extent of resection coded with biopsy as control, STR=Subtotal resection, GTR=Gross total resection. Separate analyses for necrosis (n=263), enhancement with necrosis (n=274) and edema (n=155) are all presented here. **(Left)** Fractal dimension of edema was significantly associated with overall survival, along with most variables included. **(Right)** Lacunarity of edema showed significant influence for overall survival alongside many other variables presented here.

Multivariate CPH for Progression Free Survival

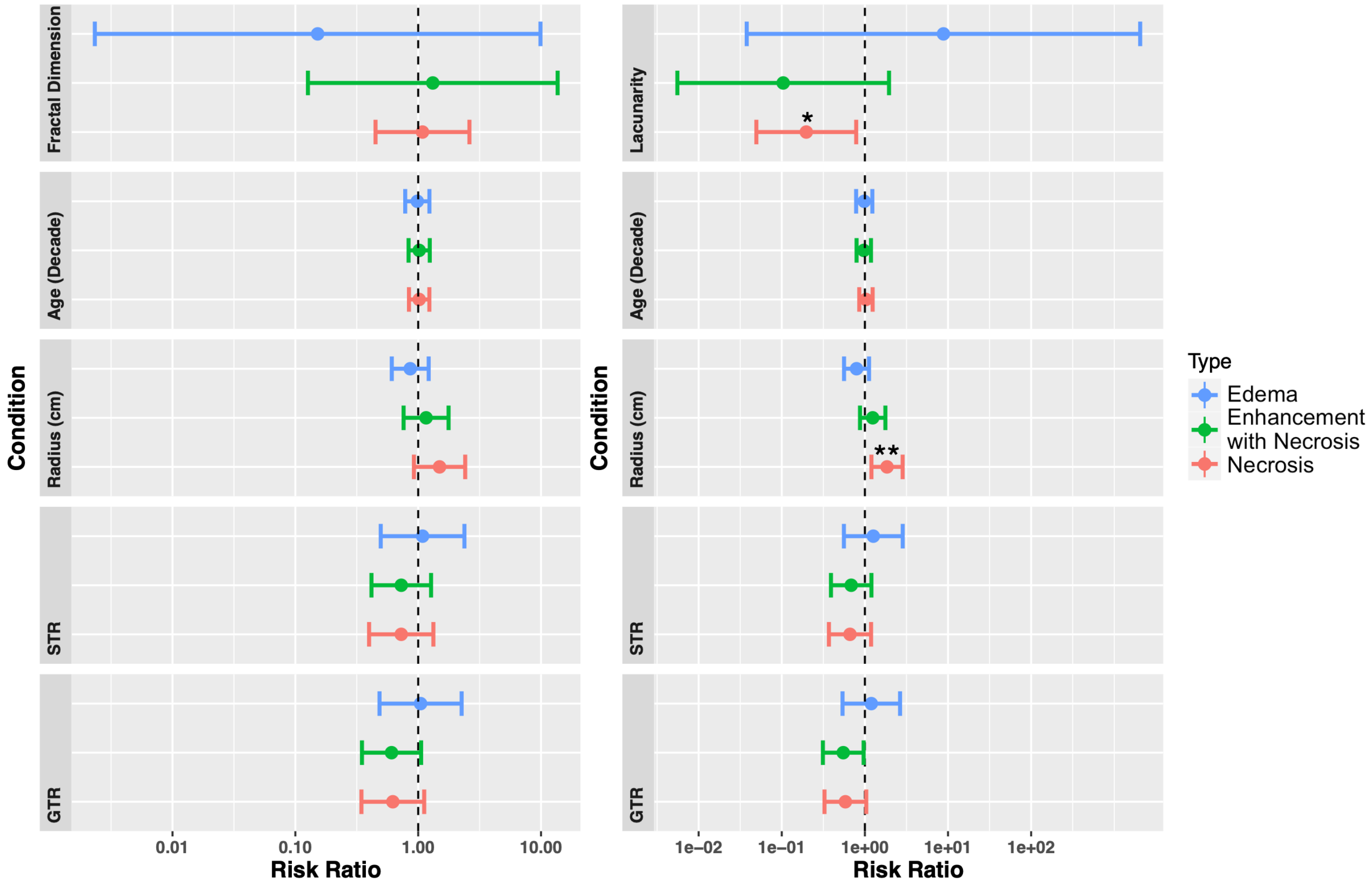

Multivariate Cox proportional hazard model for progression free survival. Extent of resection coded with biopsy as control, STR=Subtotal resection, GTR=Gross total resection. Separate analyses for necrosis (n=125), enhancement with necrosis (n=130) and edema (n=78) are all presented here. **(Left)** No variables presented as significant for progression free survival **(Right)** Lacunarity of necrosis showed a significant association with progression free survival, as did the radius of the necrotic abnormality.

Supplement 8: Image Resolution Sub-analyses

Image Resolution against Lacunarity and Fractal Dimension Values

We ran Kruskal-Wallis tests to determine the existence of a relationship between image resolution and lacunarity/fractal dimension values. We present the outcome of these 6 tests here:

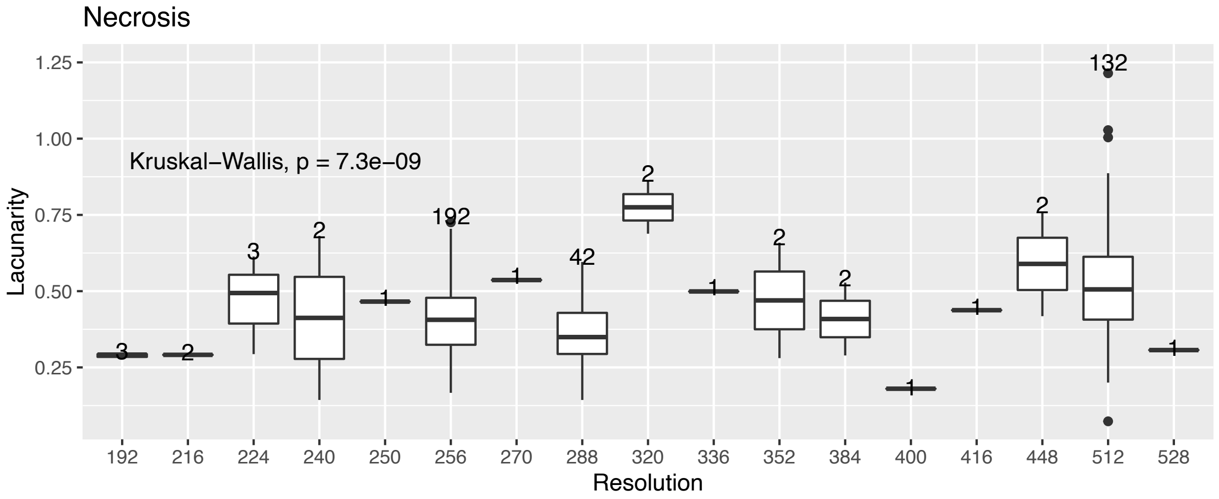

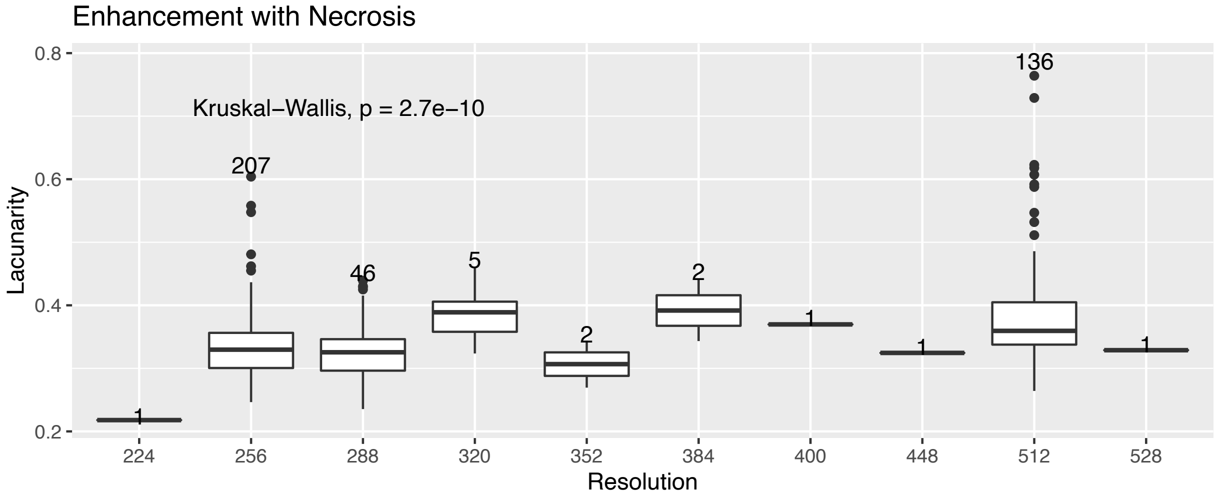

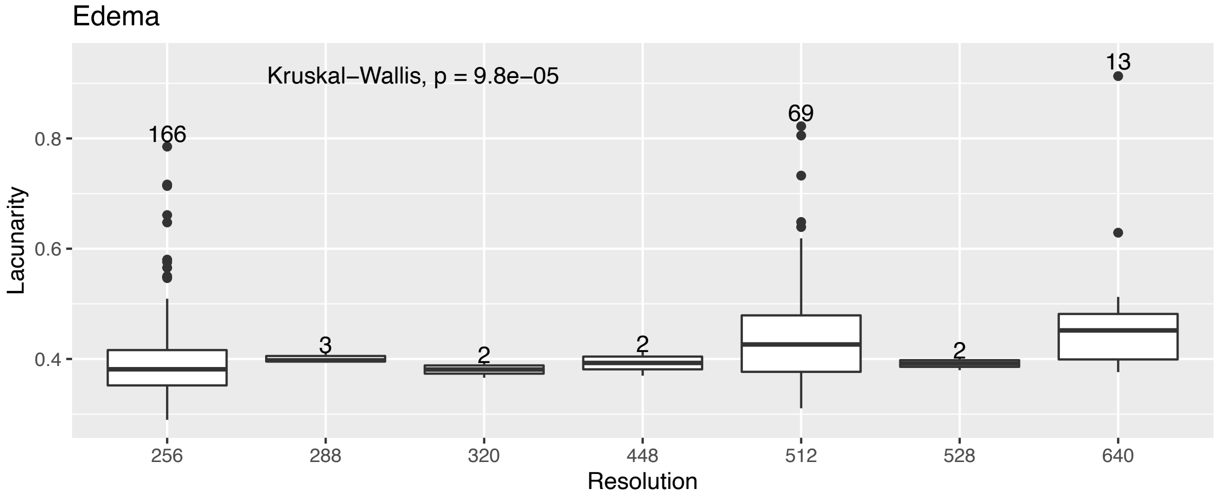

We see lacunarity is significantly associated with resolution in all three imaging abnormalities. Image count presented above each boxplot.

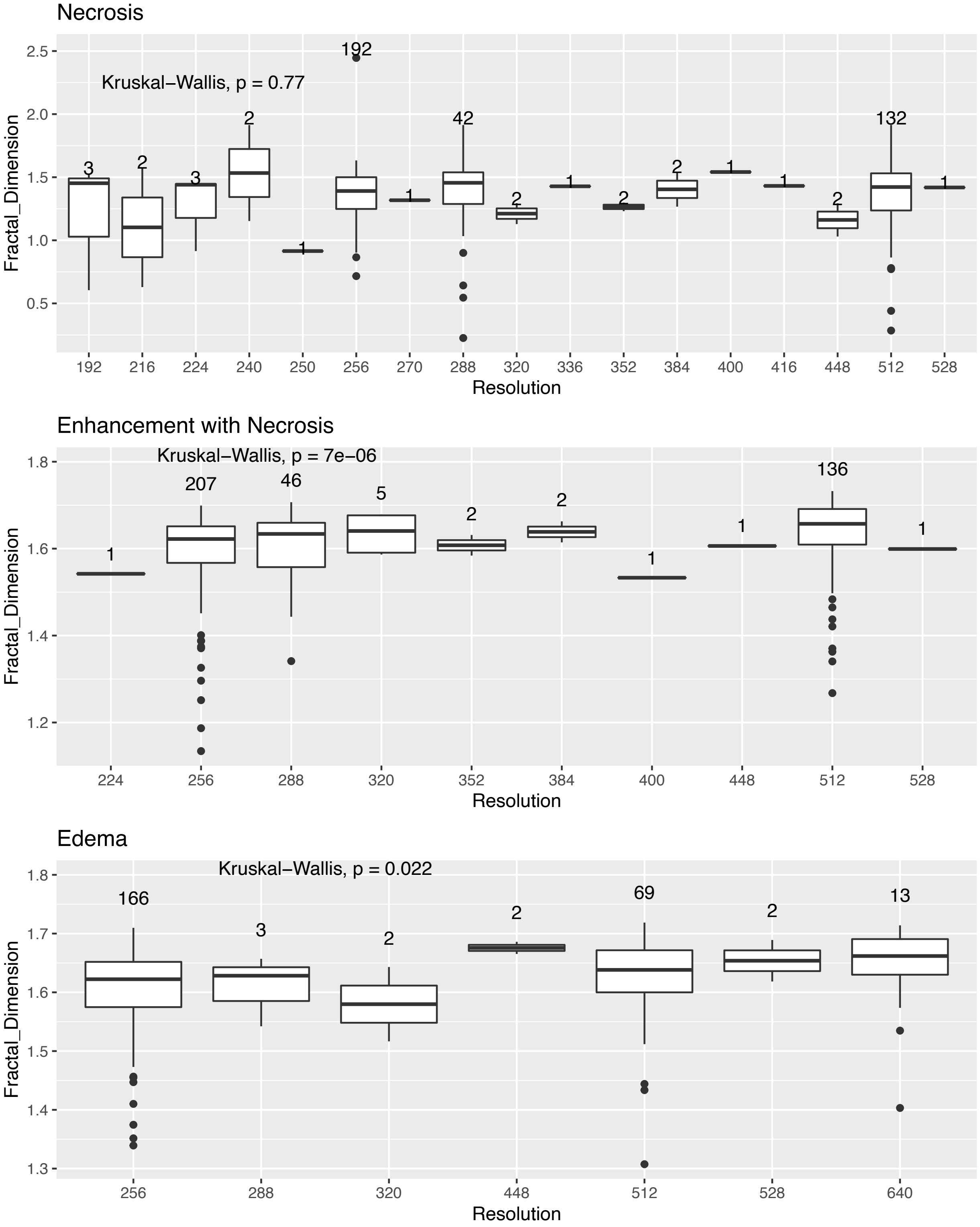

We observe significant relationships between fractal dimension and image resolution in enhancement with necrosis and edema regions, but not in necrosis. Image count presented above each boxplot

Multivariate CPH Overall Survival Plots Including Image Resolution

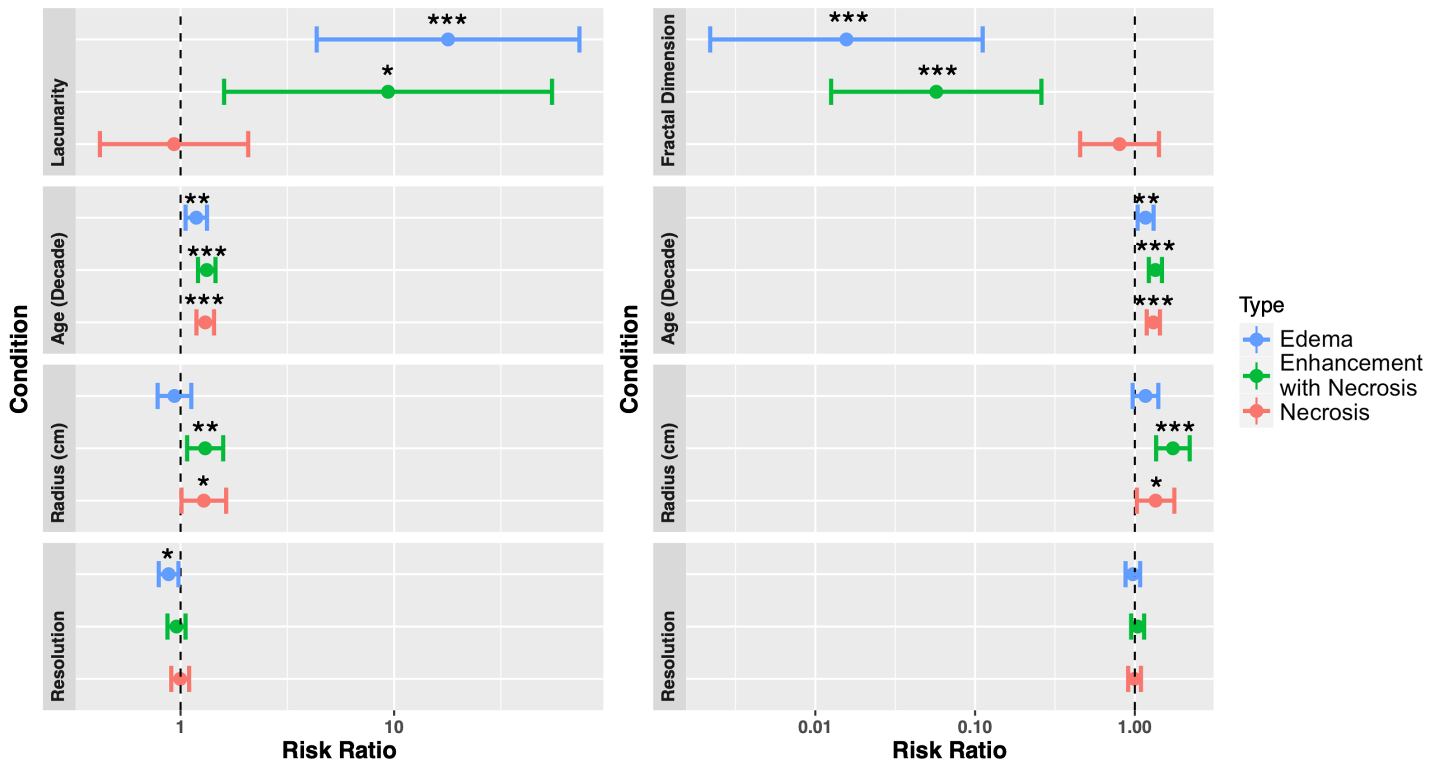

Multivariate CPH plots showing (**left**) image resolution (divided by 100 for visual scale), radius (cm), age (decades) and lacunarity **(right)** image resolution (divided by 100 for visual scale), radius (cm), age (decades) and fractal dimension. Comparing this result to **Figure 5** in the main manuscript, we see that significant lacunarity and fractal dimension results persisted through the inclusion of image resolution. Interestingly, we see T2/FLAIR resolution was significantly associated with survival in the left panel, this is in opposition to the lacunarity result (which was more significant as a result), and may point to an increase in image resolution over time alongside slight improvements in overall survival. This suggests that the relationships between resolution and imaging metrics are not the drivers of the prognostic signal we observe in the main text.

References

1. Brueckl, M., Heuer, F. and Brueckl, M.M., 2021. Package ‘irrNA’.
